# Pathogenic *Elizabethkingia miricola* Infection in Cultured Black-Spotted Frogs, China, 2016

**DOI:** 10.3201/eid2312.170942

**Published:** 2017-12

**Authors:** Ruixue Hu, Junfa Yuan, Yin Meng, Zhe Wang, Zemao Gu

**Affiliations:** Huazhong Agricultural University, Wuhan, China (R. Hu, J. Yuan, Y. Meng, Z. Wang, Z. Gu);; Hubei Engineering Research Center for Aquatic Animal Diseases Control and Prevention, Wuhan (R. Hu, J. Yuan, Z. Gu)

**Keywords:** zoonotic pathogen, *Elizabethkingia miricola*, bacillus, amphibian, frog, genetic diversity, China, bacteria, zoonoses, black-spotted frog, *Pelophylax nigromaculatus*

## Abstract

Multiregional outbreaks of meningitis-like disease caused by *Elizabethkingia miricola* were confirmed in black-spotted frog farms in China in 2016. Whole-genome sequencing revealed that this amphibian *E. miricola* strain is closely related to human clinical isolates. Our findings indicate that *E. miricola* can be epizootic and may pose a threat to humans.

*Elizabethkingia* is a genus of gram-negative, nonmotile, non–spore-forming bacilli that are ccasionally associated with human clinical infections (*1**–**6*). Although *E. meningoseptica* is the most commonly identified nosocomial pathogen of the genus (*2*), many descriptions of this species are misidentifications of *E. anophelis* and *E. miricola* (*3**–**5*). *E. anophelis*, initially isolated from the midgut of mosquitoes, caused a large outbreak centered in Wisconsin during 2015–2016 (*5*). *E. miricola* was found in 2003 in condensation water at the Mir space station (*7*). The first reported case of *E. miricola* infection was in a hematology patient in the United States in 2008 (*8*). Subsequently, *E. miricola* has been increasingly documented as causing bacteremia and sepsis in immunocompromised and immunocompetent patients, mostly in European countries (*6*). Until now, pathogenic *E. miricola* has seldom been isolated from Asia, and whether *E. miricola* can be pathogenic to animals is unknown.

The black-spotted frog, *Pelophylax nigromaculatus*, is a typical amphibian species, largely endemic to east Asia. Owing to the success of rearing it on an artificial diet, this frog has been widely farmed under special government approval as an edible animal in south-central China in recent years. In 2016, epidemic meningitis-like disease outbreaks in cultured black-spotted frogs occurred in separate farms. We identified *E. miricola* as the predominant pathogen and used whole-genome sequencing (WGS) to further characterize this Asian epizootic isolate and phylogenetically compare it with the available typical *Elizabethkingia* genomes.

## The Study

**Figure 1:**
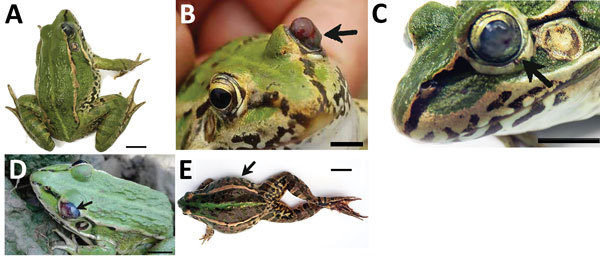
Clinical signs (arrows) in frogs with *Elizabethkingia miricola* infection in Hunan Province, China. A) Diseased frogs had neurologic signs of torticollis. B–D) Clinical signs with different appearances showing cataracts, proptosis, or hyperemia. E) Symptoms of abdominal distension. Scale bars indicate 1 cm.

Since May 2016, many black-spotted frogs in farms in Hunan Province in south-central China have experienced an emerging, contagious disease characterized mainly by severe neurologic dysfunction. The first clinical sign is intermittent swimming in circles. Thereafter, the frogs develop signs of torticollis (Figure 1, panel A), disorientation (Video), and anepithymia or meteorism (Figure 1, panel E). These signs are followed by cataracts (Figure 1, panel C); proptosis or hyperemia (Figure 1, panels B, D); agitation or lethargy; and, ultimately, death. The frogs are farmed in artificial ecologic wetlands or ponds with running water and shelter (Technical Appendix Figure 1). Most ponds in 1 farm, which share a common water supply, were infected sequentially within a short time. More than 60% of the frogs in the infected farms had signs of varying appearance, and 60%–90% of the diseased frogs died in the next few days or weeks. The disease continued until hibernation and returned the following spring.

**Video:**
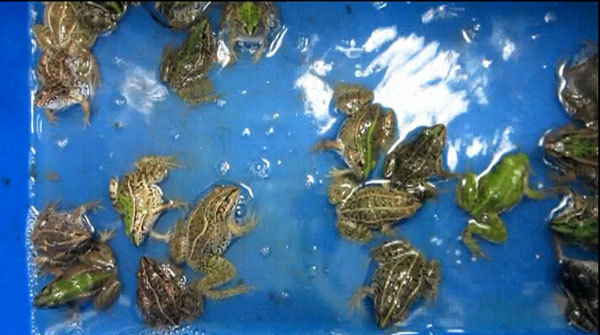
Diseased frogs sampled at Yiyang, Hunan Province, China. The diseased frogs had symptoms including neurologic signs of torticollis, disorientation, and agitation or lethargy.

During July–October 2016, we collected 213 abnormal frogs from 7 separate farms in Hunan Province, China (Technical Appendix Figure 2). Histopathologic examination showed severe meningitis with denatured, incrassate meninges. We observed inflammatory infiltrates, moderate multifocal gliosis, and perivascular cuffing in the cerebellum (Technical Appendix Figure 3). Results of the diagnostic tests for *Batrachochytrium dendrobatidis* and ranaviruses were negative (Table 1). Although we observed Myxosporidia protozoa in the gallbladder and some protists in the intestine, they were not identified as the etiologic agents, considering the proportion of infection Technical Appendix Figure 4). 

**Table 1:** Results from etiologic detection in 213 frogs collected in Hunan, China, July–October 2016*

Pathogen	Tested organ									No. positive
Skin	Liver	Spleen	Kidney	Brain	Intestine	Muscle	Gallbladder	Heart
Bacteria	NT	+	+	+	+	NT	NT	NT	NT	190†
Parasite‡	–	–	–	–	–	–	–	+	–	9
Fungus§	–	NT	NT	NT	NT	NT	NT	NT	NT	0
Ranaviruses	NT	NT	–	–	NT	NT	–	NT	NT	0

* NT, not tested; +, positive; –, negative. †Predominant bacterial infection. The results were considered positive if any one of the tested organs was positive. ‡Class Myxosporea. §*Batrachochytrium dendrobatidis.*

We confirmed bacterial infections in 190 (89.2%) of the 213 frogs; 90% were *E. miricola* according to the 16S rRNA gene sequence, which shared 99.36%–99.86% similarity with *E. miricola* DSM14571 (Technical Appendix). We selected bacterial strain FL160902, isolated from frog no. 160, as the representative isolate and conducted experimental pathogenicity testing by various infection routes, including intramuscular injection, immersion infection, and cohabitation with infected frogs. All animal handling was done in compliance with the National Institutes of Health protocols (Technical Appendix). After 2 weeks of observations (Table 2), we found that the cumulative mortality (10%–70%) increased with dose in the injection trial and that 100% of frogs exposed to *E. miricola* by immersion died. In the cohabitation studies, 30% mortality was recorded, indicating cross-infection. Koch’s postulates were satisfied by identification of isolates from dead frogs as *E. miricola*, identical to FL160902.

**Table 2:** Results of the experimental exposure of frogs to *Elizabethkingia miricola* isolate FL160902, China, 2016*

Route of infection	Concentration, CFU/mL	No. frogs per trial	Cumulative no. deaths, by days after exposure†							Mortality, %
2	4	6	8	10	12	14
Intramuscular injection‡	10^5^	10	0	1	1	1	1	1	1	10
	10^6^	10	0	0	1	1	5	5	5	50
	10^7^	10	1	3	6	7	7	7	7	70
	SPSS§	10	0	0	0	0	0	0	0	0
Immersion inoculation¶	10^6^	10	3	7	10	10	10	10	10	100
Cohabitation inoculation#	NA	10	0	0	1	3	3	3	3	30
Control	NA	10	0	0	0	0	0	0	0	0

*NA, not applicable. †Deaths after 14 d were not included. ‡Injection volume 200 μL. §An equivalent volume injection of 0.70% stroke-physiologic saline solution. ¶Immersed for 30 min in *E. miricola* suspension. #Frogs in this trial cohabited with frogs previously infected with *E. miricola*.

**Figure 2:**
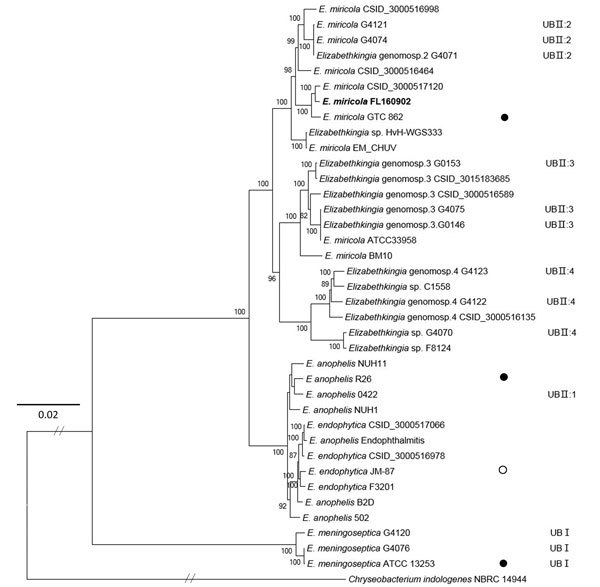
Maximum-likelihood phylogenetic tree of *Elizabethkingia miricola* FL160902 from an infected frog in Hunan Province, China, and reference genomes. The tree was constructed by using the single-copy orthologous genes of all the 38 genomes with 100 bootstrap replicates. Species identifications strictly followed the National Center for Biotechnology Information submitted names. Isolates assigned into UB groups and subgroups are according to Holmes et al. (*12*) and Bruun and Ursing (*13*).Solid circles indicate type strains; open circle indicates a former type strain. Bold indicates strain isolated in this study. Scale bar indicates nucleotide substitutions per site.

To characterize *E. miricola* FL160902, we conducted WGS with the Illumina HiSeq 2500 platform (Illumina Inc., San Diego, CA, USA), producing 2 × 150-bp paired-end reads. We assembled the trimmed reads using SOAPdenovo (http://soap.genomics.org.cn/soapdenovo.html). We constructed a phylogenetic tree (Figure 2) of orthologous genes using RAxML (*9*) with 100 bootstrap replicates to examine the evolutionary relatedness between *E. miricola* FL160902 (GenBank accession no. NHPR00000000) and other *Elizabethkingia* genomes. The results showed that FL160902 was most closely related to CSID_3000517120, a clinical isolate of *E. miricola* from the United States sequenced by the Centers for Disease Control and Prevention (CDC) (*10*), revealing the potential of *E. miricola* FL160902 for pathogenicity in humans.

Before WGS was commonly used, *E. meningoseptica* (previously *Flavobacterium meningosepticum*) was found to be separated into 2 main hybridization groups, UBI and UBII, that were ≈40%–55% interrelated; UBII could be further divided into 4 subgroups (*11*,*12*). However, because the isolates from different groups are phenotypically very similar, these genomic groups remain assigned at this time to *E. meningoseptica* (*13*). In our phylogenetic tree, UBI group *E. meningoseptica* isolates did not group with the other *Elizabethkingia* spp. and were distantly related to UBII. Considering the low DNA–DNA relatedness (<70%) between the 2 groups and phylogenomic analysis based on WGS (*3*,*11*,*12*), we propose that UBII are not *E. meningoseptica.* The UBII subgroups branching separately supports the view that they are different *Elizabethkingia* species (*3*). The UBII:1 group species *E. anophelis* and *E. endophytica* formed a clade with strong support of 100%, favoring the suggestion that *E. endophytica* is a later subjective synonym of *E. anophelis* (*14*). Our FL160902 isolate grouped with *E. miricola*, which is thought to be closely related to UBII:2 (*3*,*10*). The taxonomic status of *E. miricola* ATCC 33958 and BM10 should be reconsidered because they clustered with UBII:3 and not with UBII:2 *E. miricola* species. Our results agree with Eriksen’s conclusion about the genetic diversity in *Elizabethkingia*; a more comprehensive taxonomic system is needed to clarify the *Elizabethkingia* genus (*3*).

## Conclusions

In this natural outbreak of meningitis-like disease in cultured frogs in Hunan Province, China, in 2016, *E. miricola* was the most predominant pathogen. The neurologic signs and pathologic brain lesions suggested that *E. miricola* could break through the blood–brain barrier and damage the nervous system. The etiologic analyses combined with the results of experimental challenge support the conclusion that the *E. miricola* strain represented by isolate FL160902 is highly contagious for frogs, especially by immersion infection. We suspect that contaminated water is the primary vehicle of transmission, considering the infection assay and the epidemiology in 1 farm with different ponds. However, diverse transmission routes might be involved because there is no obvious interconnectivity among independent farms, which needs to be investigated further. Close attention should be paid to whether this disease affects the wild population of amphibians. Our results indicated the gradual expansion of its host and suggest that amphibians may serve as a reservoir for infection in humans. Black-spotted frog farming is a major aquaculture industry in south-central China; thus, animals and humans that have close contact with infected frogs should be continually monitored for emerging *E. miricola* infections, even though no human *E. miricola* infection cases were reported related to frog consumption or farming in Hunan in 2016. Our results demonstrate a contagious disease in frogs caused by *E. miricola* that poses a potential zoonotic threat to humans, generating a need for consideration of the role of *Elizabethkingia* bacteria in public health.

Technical Appendix Description of the methods used in study of *Elizabethkingia miricola* in black-spotted frogs, Hunan, China.
