# Supplementary material for: Pathogenic Elizabethkingia miricola Infection in Cultured Black-Spotted Frogs, China, 2016
Source: Emerg Infect Dis. 2017 Dec;23(12):2055–9. doi: 10.3201/eid2312.170942 (PMC5708249; doi:10.3201/eid2312.170942)
Supplement: Technical Appendix — Description of the methods used in study of Elizabethkingia miricola in black-spotted frogs, Hunan, China. [file 17-0942-Techapp-s1.pdf]

Article DOI: https://doi.org/10.3201/eid2312.170942

Pathogenic *Elizabethkingia miricola* Infection in Cultured Black-Spotted Frogs, China, 2016

Technical Appendix

Methods

Outbreak Investigations

During July–October 2016, we investigated the outbreaks of emerging, contagious disease, characterized mainly by severe neurologic dysfunction, in black-spotted frog farms in south-central China (Hunan province). Clinical observations and epidemiologic information were recorded and analyzed to determine the cause of these outbreaks. A total of 213 abnormal frogs were collected from 7 separate farms in Hunan. When the frogs were captured, potassium permanganate solution was used to disinfect equipment to avoid cross-contaminating possible pathogens between ponds.

Etiologic Examination

To figure out the etiology of this disease, we conducted bacteria isolation, microscopic parasite examination, and PCR tests for fungi and viruses in the 213 field-collected frogs. The frogs were euthanized by immersion in a 0.4% aqueous solution of MS222 followed by pithing. We then performed a routine necropsy and fixed representative tissues with 4% paraformaldehyde for histopathology. Samples taken from brain, liver, spleen, and kidney were directly streaked onto brain–heart infusion agar at 28°C for bacteria isolation. Although 190 frogs were bacteria positive, most bacterial colonies showed the same phenotype (size, color, shape). We purified 70 bacterial colonies isolated from different frogs and farms by streaking and restreaking on fresh medium, and then the 70 purified isolates were identified by Eztaxon (*1*) based on their 16S rRNA gene sequence. Among all the identified bacteria, 63 (90%) were *E.miricola*. We selected the bacteria strain FL160902, isolated from the liver of frog no. 160, as the representative strain. Its 16S rRNA gene sequence (KY461715) and *gyrB* sequence

(KY461716) were submitted to GenBank. In our etiologic examination, *Batrachochytrium dendrobatidis* (*Bd*) and ranaviruses were selected as the targeted pathogens, considering that they both contribute to global population declines in amphibians (*2*). Specifically, we tested skin samples to detected *Bd* by PCR using specific primers Bd1a and Bd2a, as described by Annis et al. (*3*). For virus detection, we extracted DNA of spleen, kidney, and muscle using a DNA extraction kit (ComWin Biotech Co. Ltd, Beijing, China). A pair of ranavirus-specific primers (*4*) and universal primers AdenoF, HVF, and ConsR (*5*) were used successively to detect ranaviruses, large DNA viruses, and adenoviruses. The primers used in our study are shown in Technical Appendix Table 1. Parenchymal tissues were checked for parasitic infections by microscopy. The results were considered positive if any of the tested organ samples was positive.

Pathogenicity Testing of *E. miricola* FL160902 to Frogs

Experimental exposure of frogs to *E. miricola* FL160902 was conducted to determine its pathogenicity. We conducted infection trials with batches of 10 healthy frogs (20.5 ± 6.7g) using various infection routes, including intramuscular injection, immersion infection, and cohabitation with infected frogs. We performed all assays in 50-L tanks supplied with wet cotton wool at 30°C. Three groups received 200 μL *E. miricola* suspensions (10^5, 10^6, 10^7 cfu/mL) by intramuscular injection, with sterile 0.70% stroke-physiologic saline solution (SPSS) injection in the fourth group as a parallel control. We performed the immersion infection by bathing frogs in 1500-mL *E. miricola* suspensions (10^6 cfu/mL) for 30 minutes. Frogs in the sixth group cohabited with 4 diseased frogs previously infected with *E. miricola* by IM injection (10^7 cfu/mL). Ten frogs in the seventh group were used as a negative control. Deaths of frogs in each of the 7 groups were recorded every day over a period of 2 weeks. Deaths after 14 days were not included in this recording. Samples from brain, liver, and kidney were tested to confirm the cause of death. *E. miricola* FL160902 was reisolated from the dying frogs, indicating that Koch's postulates had been satisfied in the challenge studies. All animal challenges in this study were carried out following the National Institutes of Health protocols and the International Society for Development Psychobiology standards (*6*).

Whole-Genome Sequencing and Phylogenetic Analysis of *E. miricola* FL160902

We extracted genomic DNA of *E. miricola* FL160902 using the SDS method (*7*). The harvested DNA was detected by agarose gel electrophoresis and quantified by Qubit (Thermo

Fisher, Waltham, MA, USA). Whole-genome sequencing was performed on the Illumina HiSeq 2500-PE150 platform with MPS (massively parallel sequencing) Illumina technology. A-tailed, ligated to paired-end adaptors and PCR amplified with a 500 bp insert and a mate-pair library with an insert size of 5 kb were used for the library construction at the Beijing Novogene Bioinformatics Technology Co., Ltd. Illumina PCR adaptor reads and low-quality reads from the paired-end and mate-pair library were filtered in the quality control step using our own compiling pipeline. All good-quality paired reads were assembled using SOAPdenovo (http://soap.genomics.org.cn/soapdenovo.html) into several scaffolds. The 15 scaffolds ranged in size from 1215 bp to 1302244 bp with a mean coverage of 160-fold. The genome of *E. miricola* FL160902 contained 4219019 bp with a GC content of 35.65%. This whole-genome project has been deposited at GenBank (BioProject PRJNA387126, BioSample SAMN07139315). The filter reads were handled by the next step of the gap closing, followed by genome components and gene function prediction.

Genomic alignments between *E. miricola* FL160902 and 37 other reference genomes were performed using the MUMmer (*8*) and LASTZ (*9*) tools. Under the analysis of ProtTest 3.2 (*10*), the best model according to BIC was JTT+I+G+F, with a confidence interval of 100.0. The phylogenetic tree of orthologous genes was constructed using the RAxML with 100 bootstrapping replicates.

References

1. Kim OS, Cho YJ, Lee K, Yoon SH, Kim M, Na H, et al. Introducing EzTaxon-e: a prokaryotic 16S rRNA gene sequence database with phylotypes that represent uncultured species. Int J Syst Evol Microbiol. 2012;62:716–21. http://dx.doi.org/10.1099/ijs.0.038075-0

2. Latney LV, Klaphake E. Selected emerging diseases of amphibia. Vet Clin North Am Exot Anim Pract. 2013;16:283–301. http://dx.doi.org/10.1016/j.cvex.2013.01.005

3. Annis SL, Dastoor FP, Ziel H, Daszak P, Longcore JE. A DNA-based assay identifies *Batrachochytrium dendrobatidis* in amphibians. J Wildl Dis. 2004;40:420–8. http://dx.doi.org/10.7589/0090-3558-40.3.420

4. Mao J, Hedrick RP, Chinchar VG. Molecular characterization, sequence analysis, and taxonomic position of newly isolated fish iridoviruses. Virology. 1997;229:212–20. http://dx.doi.org/10.1006/viro.1996.8435

5. Hanson LA, Rudis MR, Vasquez-Lee M, Montgomery RD. A broadly applicable method to characterize large DNA viruses and adenoviruses based on the DNA polymerase gene. Virol J. 2006;3:28. http://dx.doi.org/10.1186/1743-422X-3-28

6. NIH National Institutes of Health. Guide for the Care and Use of Laboratory Animals. Washington, DC: National Academies Press; 1985.

7. Chen WP, Kuo TT. A simple and rapid method for the preparation of gram-negative bacterial genomic DNA. Nucleic Acids Res. 1993;21:2260. http://dx.doi.org/10.1093/nar/21.9.2260

8. Kurtz S, Phillippy A, Delcher AL, Smoot M, Shumway M, Antonescu C, et al. Versatile and open software for comparing large genomes. Genome Biol. 2004;5:R12. http://dx.doi.org/10.1186/gb-2004-5-2-r12

9. Harris RS. Improved pairwise alignment of genomic DNA. State College, PA: Pennsylvania State University; 2007.

10. Darriba D, Taboada GL, Doallo R, Posada D. ProtTest 3: fast selection of best-fit models of protein evolution. Bioinformatics. 2011;27:1164–5. http://dx.doi.org/10.1093/bioinformatics/btr088

Technical Appendix Table 1. Primers used for virus and fungi detection

Targeted pathogen	Primer name	Primer sequence	Size	Reference
Large DNA viruses and adenoviruses	AdenoF	5-gggaattctaGAYATHTGYGGNATGTAYGC-3	1200 bp	Hanson LA et al. (*5*)
	HVF	5-cggaattctaGAYTTYGCNWSNYTNTAYCC-3	400–700 bp	
	ConsR	5-cccgaattcagatcTCNGTRTCNCCRTA-3		
Ranaviruses (Iridoviridae; Ranavirus)	RGV-P1	5′-GACTTGGCCACTTATGAC3′	531 bp	Mao J et al. (*4*)
	RGV-P2	5′-GTCTCTGGAGAAGAAGAA-3′		
Batrachochytrium dendrobatidis	Bd1a	5′-CAGTGTGCCATATGTCACG-3′	300 bp	Annis SL et al. (*3*)
	Bd2a	5′-CATGGTTCATATCTGTCCAG-3′		

Technical Appendix Table 2. Detailed results of the etiologic detection of frogs collected in 7 farms in Hunan province, China, July–October 2016

Sampling location	Sampling time	No. bacteria positive/total	No. Myxosporea positive/total	No. Bd* positive/total	No. virus† positive/total
Yiyang	Aug 9	36/42	7/42	0/42	0/42
Ningxiang	Aug 14	30/30	2/30	0/30	0/30
Changsha	Aug 14	33/35	0/35	0/35	0/35
Zhuzhou	Oct 3	17/26	0/26	0/26	0/26
Hengyang	Sept 5	21/22	0/22	0/22	0/22
Shaoshan	Jul 20	26/31	0/31	0/31	0/31
Wugang	Jul 21	27/27	0/27	0/27	0/27

Batrachochytrium dendrobatidis.
†Ranaviruses or large DNA viruses.

Technical Appendix Table 3. Results of the identified bacteria isolated from different organs

Organ	Identified isolates	*Elizabethkingia miricola*	Proportion
Brain	23	20	86.9%
Liver	16	14	87.5%
Spleen	13	12	92.3%
Kidney	18	17	94.4%
Total	70	63	90%

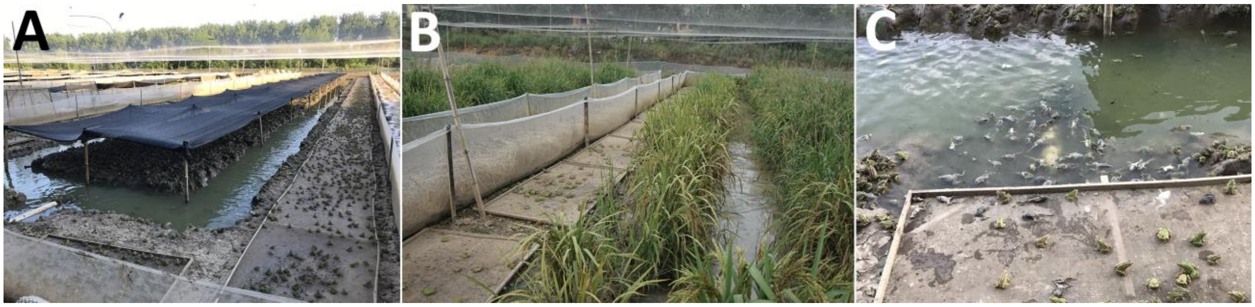

Technical Appendix Figure 1. Different frog farms in Hunan Province, China. A) Ponds with running water and shelter. B) Artificial ecologic wetlands with rice. C) Mass mortality because of disease.

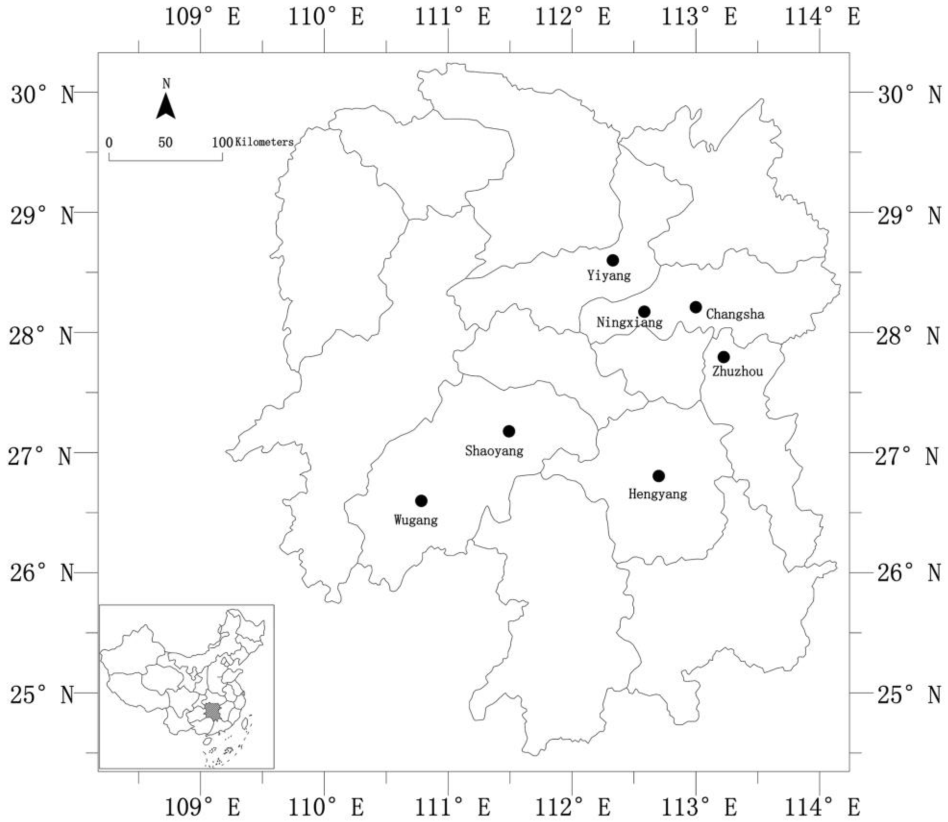

Technical Appendix Figure 2. Map of sample locations of diseased frogs in Hunan Province, China.

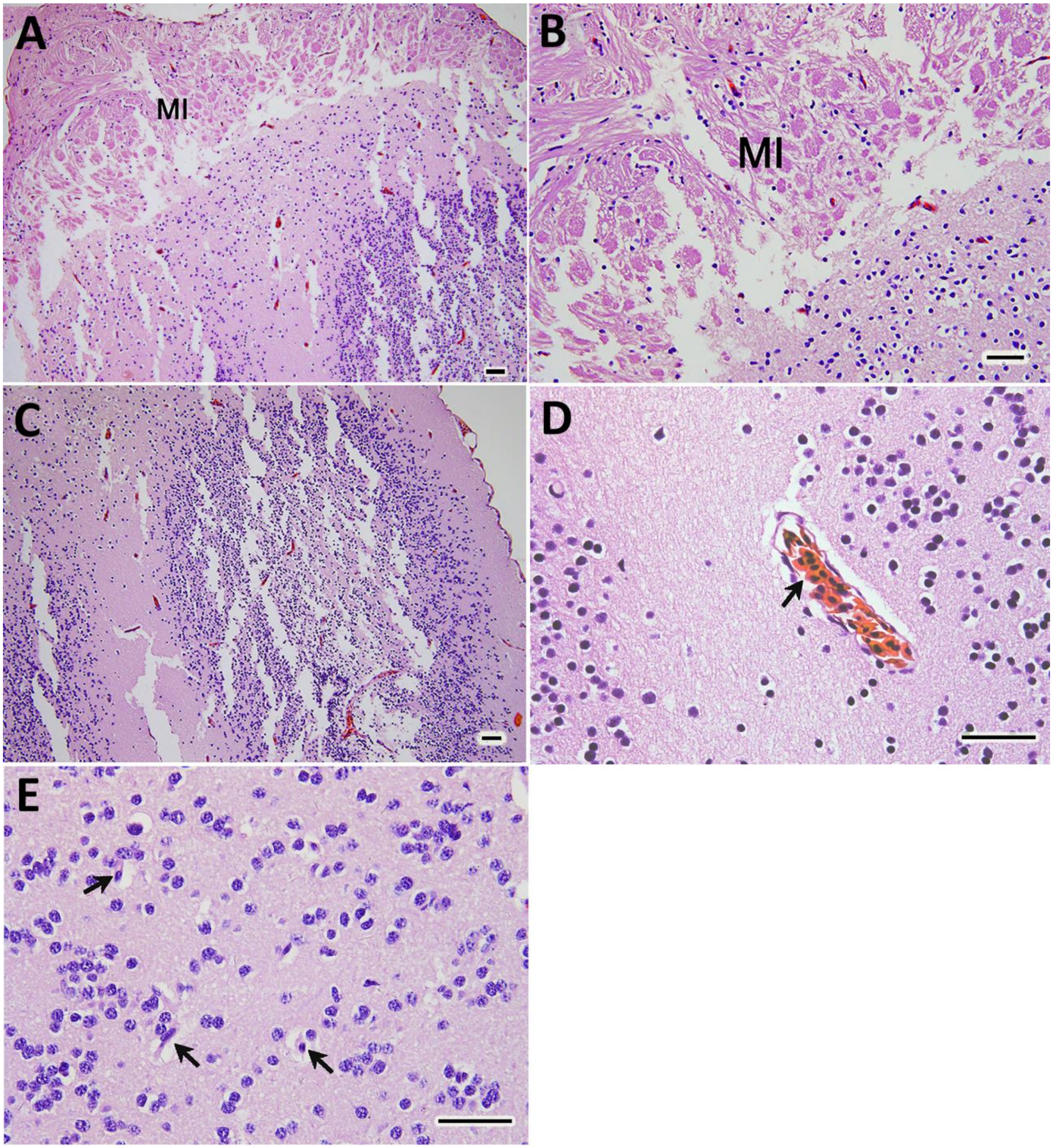

Technical Appendix Figure 3. Pathologic brain lesions of diseased frogs in Hunan Province, China. A) Meningitis with meninges incrassation (MI). B) Denaturation in the incrassated meninges. C) Inflammation with lymphohistiocytic infiltration in cerebellum. D) Expanded perivascular space in brain parenchyma (single-arrow). E) Degenerated and necrotic nerve cell in ectocinerea (single arrow). All scale bars indicate 50 μm.

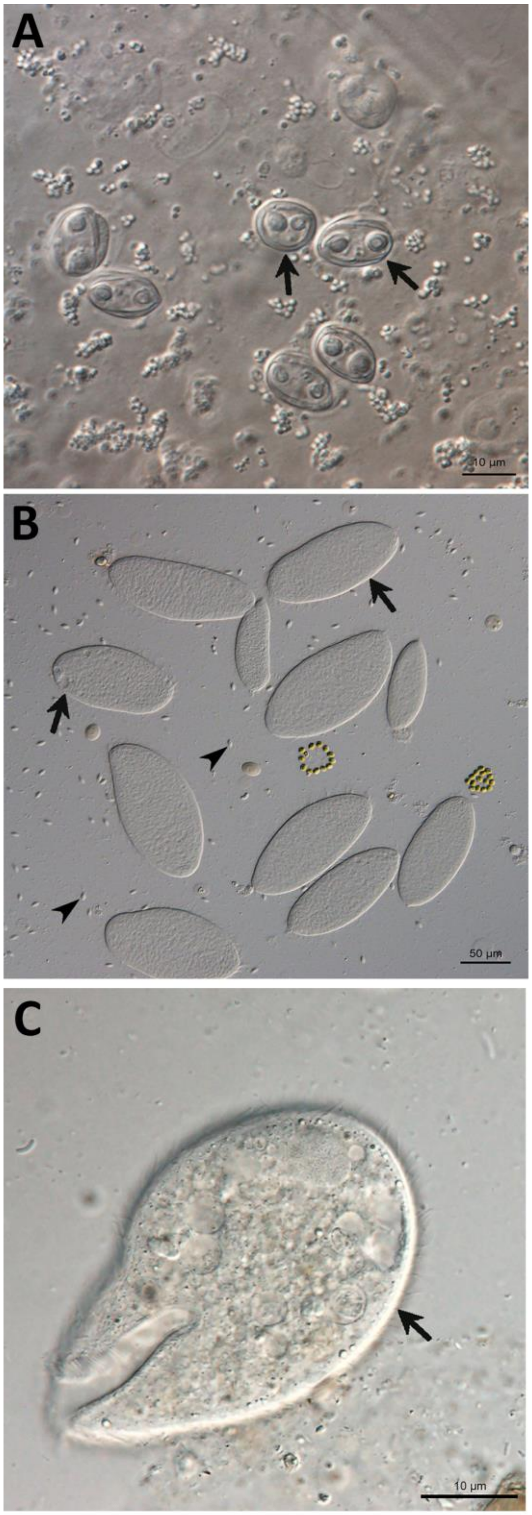

Technical Appendix Figure 4. Typical microorganisms observed in optical microscopy of diseased frogs in Hunan province, China. A) Parasitic myxospore observed in gallbladder; bar, 10 μm. B) Intestinal protist *Opalina* sp. (single arrow) and unknown flagellates (arrowhead); bar, 50 μm. C) Intestinal *Blantidium* sp.; bar, 10 μm.
